# Country-Brand Fit: The Effect of COO Stereotypes and Brand Positioning Consistency on Consumer Behavior: Evidence From EEG Theta-Band Oscillation

**DOI:** 10.3389/fnins.2022.901123

**Published:** 2022-05-23

**Authors:** Ailian Wang, Dong Lyu, Yunlu Liu, Jiaoyang Liu, Li Gao, Jia Jin

**Affiliations:** ^1^Laboratory of Applied Brain and Cognitive Sciences, School of Business and Management, Shanghai International Studies University, Shanghai, China; ^2^Research Center for Intelligent Society and Governance, Research Institute of Interdisciplinary Innovation, Zhejiang Lab, Hangzhou, China; ^3^Academy of Neuroeconomics and Neuromanagement, Ningbo University, Ningbo, China; ^4^SILC Business School, Shanghai University, Shanghai, China

**Keywords:** country-of-origin stereotypes, brand positioning, cognitive consistency theory, EEG, theta-band oscillation, purchase intention

## Abstract

Grounded on the cognitive consistency theory, this paper adopts the prime-probe paradigm and Electroencephalography (EEG) experiment to examine the impact of country-of-origin (COO) stereotypes-brand positioning congruence on consumer behavior, the boundary effect of brand positioning strategy, as well as the underlying cognitive mechanism. Behaviorally, consumers show a higher purchase intention in the congruence condition. Moreover, this congruence effect of purchase intention can be found for competence brand positioning strategies rather than warmth brand positioning strategies. At the brain level, we found that compared with the congruence condition, the incongruence condition enhances consumers' cognitive conflict, reflected in enhanced frontal theta-band oscillation. Furthermore, the cognitive conflict effect is accentuated in the competence positioning strategy condition rather than the warmth strategy positioning condition, confirming the boundary effect of brand positioning strategy from the brain level. These findings provide neural evidence that the congruence between COO stereotypes and brand positioning influences consumer purchase behavior, reveals a boundary effect in the COO stereotype-brand positioning congruence, and highlights the importance of the competence dimension. Finally, the theoretical and practical implications are discussed.

## Introduction

Steadily accelerating globalization has profoundly impacted both consumers and companies, and maintains its continuity in marketing activities (Makrides et al., 2021; Shams et al., 2021). Notably, the influence of country image on perceptions of products originating from different countries, a phenomenon is known as the country-of-origin (COO) effect, has been long investigated in international marketing literature. COO stereotypes are an oversimplified set of beliefs about nations and their members (Halkias et al., 2016), which are formed through direct or indirect experience with the country and are stored as cognitive elements in a consumer's memory (Maheswaran, 1994). For example, the informational cue “made in Germany” signals several internal and external cues like design, reliability, and price level. Maher and Carter (2011) considered the function of COO as an extrinsic cue that influences consumer choices when they decide to choose between domestic vs. foreign products (Maher and Carter, 2011). Chen et al. (2014) found that negative (vs. positive) country-based emotions lead to less (vs. more) favorable product evaluations (Chen et al., 2014). A consistent conclusion across the COO studies is that consumers organize their associations with countries into mental schemas, which influence brand evaluations (Kotler and Gertner, 2002; Magnusson et al., 2018). Consequently, researchers have shown growing interest in the products' COO effect on consumer attitudes (Fischer and Zeugner-Roth, 2017; Barbarossa et al., 2018; Magnusson et al., 2018; Meese et al., 2019). Noteworthily, the COO stereotype is difficult to change because of consumers' long-term and stable brand image (Clifton, 2017), which has brought considerable resistance for brands that attempt to enter the markets of other countries.

Based on previous studies, a brand can be effectively positioned to associate with its home country's personality stereotype. For instance, Magnusson et al. (2018) found that brands are evaluated more favorably when positioned in a manner congruent with their home country personality stereotypes than when their brand positioning is incongruent with such stereotypes (Magnusson et al., 2018). Besides, Meese et al. (2019) explored the relationship between country-of-origin and brand positioning in the context of the high-involvement service of health care. Their findings showed that matching the brand positioning of international healthcare organizations with their country images is a crucial way to attract international patients. Aligning the brand positioning with the COO stereotype is seemingly beneficial to the consumer's brand evaluation. Moreover, several studies have used the cognitive consistency theory to explain the benefit of COO stereotypes and brand positioning congruence. As proposed by Festinger (1957), cognitive consistency theory assumes that consumers are predisposed to prefer elements that are cognitively consistent with their existing knowledge (Festinger, 1957). From this, consumers are predisposed to prefer new information (i.e., brand positioning information) cognitively consistent with their current knowledge (i.e., country schema) that reinforces previous stereotypes (Festinger, 1957; Magnusson et al., 2018). In contrast, brand positioning-country incongruence contradicts expectations and produces cognitive conflict and imbalance, thus engendering unfavorable brand evaluations. As such, we first posited that consumers would show higher intention to purchase products whose COO stereotypes are congruent with the brand positioning instead of products that are incongruent.

As for the classification of COO stereotypes, in this study, we employ the Stereotype Content Model (SCM) (Özsomer, 2012; Diamantopoulos et al., 2017; Fiske et al., 2018; Micevski et al., 2020), which posits that individual judgments about nations are consequences of the social structural relationships between national groups and mainly refer to two independent, stereotypical dimensions: competence and warmth (Fiske et al., 1999; Bourdin et al., 2021). In the marketing domain, COO competence refers to consumer cognitive appraisals about a country's capability, efficacy, and efficiency (Barbarossa et al., 2018). For example, one of the reasons BMW is perceived as a competent brand maybe because it conveys the stereotype of German competence (Diamantopoulos et al., 2011). COO warmth refers to consumer cognitive appraisals about a country's friendliness, cooperativeness, and trustworthiness (Barbarossa et al., 2018). Zara may benefit from its origin's (i.e., Spain) relatively high warmth by establishing a more consonant country stereotype; for example, by stressing Spanish people's friendliness and “joie de vivre” in its brand communications (Diamantopoulos et al., 2021). Researchers suggested that individuals perceive countries to have different degrees of competence and warmth (Cuddy et al., 2008) and that they unintentionally convey (Herz and Diamantopoulos, 2013) such perceptions to products originating from those countries. In turn, these perceptions (including emotion and cognition) impact consumer responses toward these products (Chattalas, 2015).

For the brand positioning, following Heinberg et al. (2017), we define it as the utilization of promotional messaging to establish key associations in customers' minds (Heinberg et al., 2017). Brand positioning is key to shaping a brand personality attributes, as they convey symbolic and cultural aspects that enable consumers to resonate with the brand in a human-like way (Aaker et al., 2001). Recent studies on brand positioning and its congruence with country personality focused on two brand positioning strategies: competence and warmth (Magnusson et al., 2018; Kervyn et al., 2022). Given that firms are unlikely to use overtly negative traits (e.g., wickedness) to position their brands and the fact that the current study focuses on COO stereotypes and brand positioning congruity, we will examine positive brand positioning and adopt these two kinds of brand positioning that correspond to the COO stereotypes. Competence-positioning brands emphasize the competence aspect including the use of competence-oriented slogans (e.g., Audi: “Advancement Through Technology”), while warmth-positioning brands communications should be portraying brand with good intentions (i.e., being nice, warm and kind) (Kolbl et al., 2020). It may be easy to assume that competence is a favorable brand attribute that all brands should tout in some industries (Aaker et al., 2012; Magnusson et al., 2018). According to cue-diagnosticity theory, consumers face multiple cues in assessing a product, such as price, brand reputation, advertising, or product reviews, and the cues provide information on product quality that are not observable until actual consumption (Purohit and Srivastava, 2001). In this study, we consider the competence-positioning dimension as high-scope cues as they meet two characteristics of high-scope cues. First, competence-positioning highlights brand ability, which is directly linked to product quality and influences consumers' product evaluation. Second, the valence of competence-positioning is stable and will not be changed within a short time (Akdeniz et al., 2013). In comparison, the warmth-positioning dimension may serve as low-scope cues by virtue of the emotional function it conveys and the indirectness between it and product quality. Besides, Chen et al. (2014) argues that warmth levels are perceived as non-diagnostic for the quality or performance of country products in terms of international marketing (Chen et al., 2014). Thus, we assume that competence-positioning is a more prominent factor in shaping a brand, and the congruence effect is expected to be more pronounced with competent brand positioning than the warm one. In other words, a larger difference in purchase intention is expected to be observed between competence and warmth stereotype countries in the competent brand positioning condition rather than in the warm brand positioning condition.

Prior studies regarding COO stereotypes heavily relied on consumer self-reporting, with little concern for the effect from the perspective of implicit consumer perception since the cognitive processes are more implicit and difficult to measure directly. Lack of physiological-level cognitive evidence hinders our understanding of the brand positioning-country personality congruence, as the COO stereotype is widely recognized as a cognitive factor. Beyond the behavioral measures employed in previous studies regarding COO stereotypes and brand positioning, the Electroencephalography (EEG) method is introduced in our current study. During the past few decades, rapid advancements have been witnessed in neuroscience technology, and neuroscientific tools (especially the EEG method) have recently been adopted in the marketing domain since they can provide a more direct approach for measuring consumers' cognitive processes when engaged in tasks (Shiv and Yoon, 2012; Yoon et al., 2012). Hence, the current study intends to adopt EEG to explore the cognitive processes of the congruence effect between COO stereotype and brand positioning to better understand its underlying mechanism.

In previous neuromarketing studies, theta band observations are frequently made to examine consumers' cognitive processes in response to marketing stimuli (Telpaz et al., 2015; Diao et al., 2021). The theta rhythm of human scalp electroencephalogram (EEG) is usually defined at 4–8 Hz (Jia et al., 2020). It is primarily generated in structures of the limbic system (e.g., the hippocampus and the cingulated cortex) and found in the prefrontal cortex (Nigbur et al., 2011). Frontal theta band has been recognized for a wide span of cognitive functions such as focused attention, novelty encoding, working memory loading, and top-down cognitive control (Cavanagh and Frank, 2014). Notably, regarding its role of cognitive control in conflict monitoring, previous stereotype-related study has shown that the frontal midline theta-band are thought to reflect cognitive control. For instance, Jia et al. (2020) found that an event-related synchronization accompanies the enhanced performance of stereotype inconsistent trials on the frontal theta oscillation (Jia et al., 2020). A handful of neuroscience studies also demonstrated that the stereotype control shares a similar conflict interference mechanism with the domain-general cognitive control of the prefrontal cortices (Knutson et al., 2007; Cattaneo et al., 2011). Since a larger magnitude of theta-band activity may suggest an inconsistent conflict between COO stereotypes and brand positioning, we predict that the enhanced theta-band activity can be observed when brand positioning and COO stereotypes are incongruent, compared with the congruent condition. Similarly, according to the behavioral hypothesis of the conditional effect, we also posit that consumers' cognitive conflict toward competent COO products with a warm brand positioning may be attenuated, leading to a smaller theta-band difference between competence and warmth stereotype countries than in the competent brand positioning.

To sum up, this paper intends to conduct an EEG experiment to explore the impact of COO stereotype-brand positioning congruence on consumer behavior and the underlying mechanism by uncovering its cognitive processing. Moreover, we also aim to examine the conditional effect by focusing on specific brand positioning strategies. Firstly, we assume that the incongruence between COO stereotypes and brand positioning reduces consumers' purchase intention compared with the congruence condition, as reflected in a stronger theta-band oscillation of the cognitive process. Secondly and more remarkably, a disparate consistency effect in different brand positioning strategies is foreseeable.

## Materials and Methods

### Participants

Before recruiting subjects, we conducted a priori power analysis for a 2 × 2 within-subjects, repeated-measures analysis of variance (ANOVA) using G^*^Power 3.1 software to estimate the sample size (Faul et al., 2007). Some parameters on the panel are set as follows: a power with 0.8, an effect size of 0.40 (a large effect size), and an alpha of 0.05. The result showed a sample of 19 participants was required. In general, we need at least 19 subjects in this study, and the more, the better.

The participants of the current experiment consisted of 39 volunteers (19 males, 20 females). All the participants did not have any history of neurological or mental diseases. They are native Chinese speakers who had normal or corrected to normal vision. Their age ranged from 18 to 26 years, with a mean age of 21.15 years (S.D. = 1.20). Each participant provided written informed consent before the formal experiment started. It is also following the ethical standards of the institutional research committee and with the 1964 Helsinki Declaration and its later amendments or comparable ethical standards (World Medical Association, 2013) and was approved by the school internal review committee.

### Materials

This experiment used the prime-probe paradigm, in which the prime stimuli (COO information) and probe stimuli (brand positioning strategy) were presented sequentially during one trial procedure. Experimental materials comprised 160 stimuli, which consisted of 2 kinds of the country (competence- vs. warmth- COO stereotype) × 2 kinds of brand positioning strategies (competence vs. warmth). Below we describe the stimuli construction in detail.

As for the country selection, we aim to choose two countries that the general consumer considers to represent these two COO stereotypes (competence vs. warmth) best. First, we randomly interviewed 20 college students and asked them to list the three countries which they thought to have the most competent/warm country image in their minds, respectively. Preliminary results suggested that Germany and America are stereotypical competence countries, while France and Italy are stereotypical warmth countries. Second, we recruited another group of participants (*N* = 35) and asked them to complete the national stereotype measurement questionnaire [7-point Likert scale, e.g., How warm are members of Germany, adapted from Fiske et al. (2018)] and rate their familiarity with these countries (7-point Likert scale). Paired samples *t*-test results indicated that the national stereotype toward Germany [*Mc* = 5.789, *S.D*. = 0.727, *Mw* = 2.679, *S.D*. = 1.302, *t*_(1,34)_ = −8.546, *p* <0.001], America [*Mc* = 5.788, *S.D*. = 0.762, *Mw* = 2.678, *S.D*. = 0.989, *t*_(1,34)_ = −12.649, *p* <0.001], Italy [*Mc* = 3.159, *S.D*. = 1.33, *Mw* = 5.271, *S.D*. = 1.015, *t*_(1,34)_ = 6.391, *p* <0.001] and France [*Mc* = 3.126, *S.D*. = 1.223, *Mw* = 5.064, *S.D*. = 1.038, *t*_(1,34)_ = 5.445, *p* <0.001] all had significant differences in warmth and competence dimensions. A further paired samples *t*-test for familiarity showed that subjects were more familiar with Germany than America [*M*_Germany_ = 5.171, *S*.*D*._Germany_ = 1.581, *M*_America_ = 4.657, *S*.*D*._America_ = 1.161, *t*_(1,34)_ = −2.052, *p* = 0.048], and more familiar with Italy than France [*M*_France_ = 4.000, *S.D*. _France_ = 1.434, *M*_Italy_ = 4.857, *S*.*D*._Italy_ = 1.166, *t*_(1,34)_ = −2.533, *p* = 0.016]. Thus, based on the screening process above, we deemed competence (Germany) and warmth (Italy) as the two most salient descriptors of those countries.

Furthermore, for the selection of products, since our research purpose and research objects are independent of the product type, we aim to screen out products with the most neutral attribute preferences (neither like nor dislike, 4 points on a 7-point Likert scale) and less inter-individual variations. Initially, we selected ten kinds of daily-use products that can be purchased and familiarto the student participants: Pen, Bluetooth speaker, Bluetooth headset, Electric toothbrush, Chocolates, Pillows, Backpack, Honey, Sunglasses, and Records. Then, we recruited 20 subjects (not participating in the formal experiment later) to measure their preferences toward these products (7-point Likert scale, e.g., How much would you say you like Bluetooth speaker). Results showed that the preference for the Pen (*M* = 4.13, *S.D*. = 0.916), Bluetooth speaker (*M* = 4.24, *S.D*. = 0.583), Bluetooth headset (*M* = 4.05, *S.D*. = 0.783), an Electric toothbrush (M = 4.34, *S.D*. = 0.978) were the most neutral, with only minimal variance. Further one-way analysis of variance showed no significant difference in the subjects' preference for these products [*F*_(3,76)_ = 0.270, *p* = 0.847]. For standardization, we firstly downloaded pictures of these four kinds of products as stimuli for the formal follow-up experiments. Then, all images were edited using Photoshop 13.0 (Adobe Systems Incorporated, San Jose, CA, USA). Eventually, we created a standard product image library of 40 images. Finally, for the selection of brand positioning slogans, we invited five marketing experts to screen out 15 advertising slogans from the Internet for each product in each brand positioning (i.e., competence vs. warmth), and a total of 120 slogans were obtained. In the second round of selections, 40 college students from different majors were recruited to choose 6 slogans out of every 15 advertising slogans with the most distinctive brand positioning characteristics (e.g., Please select 6 slogans from the following 15 slogans that best highlight the brand's warmth personality). Then, 48 slogans, including 6 slogans for each brand positioning of 4 products, were left as the final stimuli. Table 1 shows some of the brand positioning advertising slogans. All the slogans consist of an average of 8 Chinese characters in SimSun 12 font. Each stimulus image with a gray background and a product image (randomly selected from the candidate product image library) next to the slogan was digitized into 360 × 270 pixels. All images of the stimulus material had the same brightness and contrast.

**Table 1:** Examples of the brand positioning advertising slogans.

**Product**	**Competence**		**Warmth**	
	**Chinese**	**English**	**Chinese**	**English**
Pen	创新阻尼设计	Innovative damping design	书写生活温暖无界	Writing life, with warmth but without boundaries
	尖端含有钛金粒	Titanium-contained tip	品味优雅服务舒心	Elegant taste, considerate service
	高强度ABS材质笔杆	Tough ABS-made barrel	书写动人回忆	To write down touching memories
Bluetooth speaker	主动式空间补偿技术	Active volumetric compensation technology	夜间模式贴心守候	Intimate guard of night mode
	智能FOD金属异物检测技术	Intelligent FOD (foreign object detection technology)	音色悦耳温暖通透	Melodious, warm and penetrating
	汽车烤漆工艺	Automotive painting techniques	小巧便捷随身享受	compact, handy and portable
Bluetooth headset	高分子复合振膜材料	High polymer composite diaphragm material	享受酣畅体验	Delightful use
	智能双处理芯片技术	Intelligent dual-processing chip technology	轻盈柔软而坚韧	Light, lithe and tough
	电容式触控技术	Capacitive touch technology	贴耳设计呵护更贴心贴心贴心	Better experience from ear-fitting design
Electric toothbrush	高精度加速传感器	High-precision accelerometer	关爱你的微笑	Care for your smile
	声波气旋震动	Sonic cyclonic vibration	流动洁力迷人笑容	Fluid cleansing for glamorous smile
	无境动力输出系统	Endless power output system	贴心专属时尚	enjoyable, suitable, fashionable

### Procedure

Participants were seated comfortably in front of a computer screen in an electrically isolated room. The experimental stimuli were presented in the center of a computer screen at a distance of 100 cm, with a visual angle of 8.69° × 6.52° (15.2 × 11.4 cm, width × height). We used the E-prime 3.0 software package (Psychology Software Tools, Pittsburgh, PA) for the stimuli presentation, triggers, and response recording. A keypad was provided for subjects to make choices. The experiment consisted of four blocks, and each block contained 40 trials. Counterbalancing of blocks was manipulated among the subjects. Practice trials were administered before the formal experiment.

**Figure 1:**
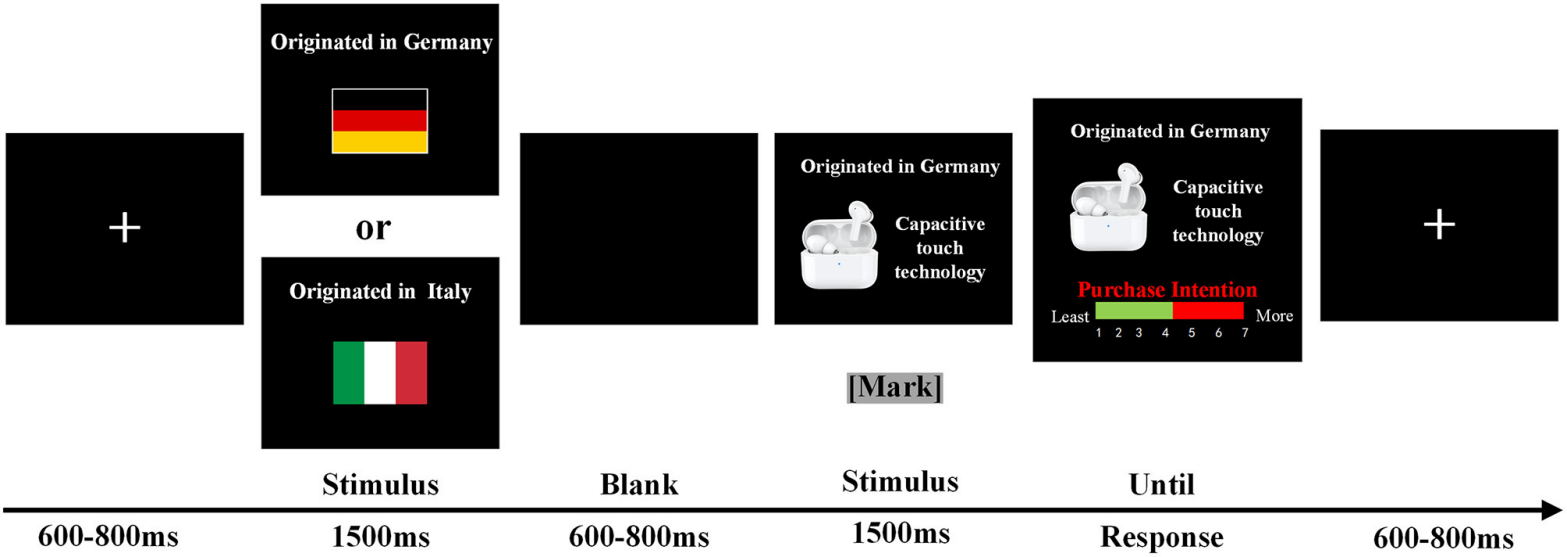
A single trial in the purchase intention task. Each trial began with a fixation cross. Participants viewed the country information for 1,500 ms. After a blank screen of 600–800 ms, the brand positioning information was presented for 1,500 ms. Then, they were asked to give the purchase intention by pressing the corresponding button. EEGs were recorded from the participants throughout the experiment.

A single trial is illustrated in Figure 1. A fixation appeared at the beginning of each trial for 600–800 ms on a black screen. The COO information was subsequently presented for 1,500 ms, including the country name and flag. After the interval for 600–800 ms, brand positioning information was shown for 1,500 ms, marked for EEG analysis. Then, participants were asked to report their purchase intention to the current product on a 7-point scale (key 1 for decrease, key 3 for increase, and key 2 for confirmation).

### EEG Data Recording and Analysis

In the EEG data collection stage, the parameters re set at bandpasss 0.05–100 Hz, sampling rate 500 Hz with a SynAmps2 amplifier system, and Curry 8 recorder software (Neuroscan Inc., Herndon, VA, USA). The EEG cap included 64 Ag/AgCl scalp electrodes arranged based on the international 10–20 system. An electrode between PFz and Fz on the forehead was regarded as the ground electrode, and the online reference electrode was located in the left mastoid. The vertical electrooculogram (EOG) was recorded by electrodes placed supra-and infra orbitally to the left eye, and the horizontal EOG was recorded from the left and right orbital rims. The formal experiment began after all electrode impedances were <5 kΩ.

EEG data preprocessing was performed using the EEGLAB (Delorme and Makeig, 2004) toolbox version 14.1.1 for MATLAB (R2013a, The MathWorks, Inc., Natick, MA). EEG data were bandpass filtered to a range of 0.1–30 Hz, re-referenced to the average of the left and right mastoids, and epoch from −1s to +2s surrounding the probe screen onset. Further, we conducted the independent component analysis. ICs representing eye blinks or other artifacts were removed from the EEG data (mean number of removed ICs across subjects = 2.026, *S.D*. = 0.279). Next, we used the CSD toolbox [spline flexibility (m) = 4, λ = 1.0 × 10^−5^, with 50 iterations for all EEG scalp sites] to carry out the current source density (CSD) transformation (Kayser and Tenke, 2006). The CSD transformation was used to minimize the volume conduction effect and identify the electrodes sufficiently representing task-relevant cognitive processes (Dippel et al., 2017).

Based on previous studies (Kubota et al., 2001; Cohen, 2014; Diao et al., 2021), we selected theta-band activity (4–7 Hz), 250–350 ms, and frontal-center electrodes (i.e., Fz, F1, F2, FCz, FC1, FC2, Cz, C1, and C2) as the ROI for the average power analysis. All analyses were implemented in MATLAB. To compute the Short Time Fourier Transform (STFT, $\overset{n-1}{\underset{n=0}{\sum }}s\left(n+mN^{\prime }\right)w\left(n\right)e^{-j\frac{2\pi }{N}nk}$), the signal is partitioned into several segments of short-time signals by shifting the time window with some overlapping (Zabidi et al., 2012). A hamming window technique is then applied to maintain the continuity between the beginning and the last points in the frames, preventing the leakage effect in the spectrum. In this analysis, N represents window segment length, *N*′ represents the shifting step of the time window, *w*(*n*) represents the window method of an N point sequence. Then, the spectrogram was built from a sequence of spectrum (windows that applied with FFT) by stacking them together in time and compressing the amplitude axis into a “contour map.” We set frequency increases from 1 to 30 Hz in 30 logarithmically spaced steps, the time to correspond to 2 s per time point, so there are 1,500 time points and normalized the oscillatory power in decibel (dB) scale [conversion equation: dB power = 10^*^log10 (power/baseline)] to ensure that the oscillatory power across all conditions and participants were comparable, where the −350 ms to −150 ms pre-stimulus was taken as the baseline activity.

The purchase intention is normally distributed (Kolmogorov–Smirnov's *D* = 0.892, *p* = 0.403). In the formal data analysis section, we analyzed behavioral and neural data in two parts. Firstly, to test the consistency effect, we reinvented the congruence (the average of German-competence and Italian-warmth positioning) and incongruence (the average of German-warmth and Italian-competence positioning) conditions. Then we conducted a paired samples *t*-test on the congruence and incongruence of purchase intention and averaged theta-band activities, respectively. Secondly, to examine the conditional effect of COO stereotypes effect, within-subjects repeated-measures ANOVA for the purchase intention and averaged theta-band were performed with COO stereotypes (competence vs. warmth) × brand positioning (competence vs. warmth). We further conducted a simple effect analysis when the interaction effect between factors was significant. In all ANOVA, effect sizes were reported by partial eta-squared ($\eta_{p}^{2}$), of which 0.05 indicates a small effect, 0.10 a medium effect, and 0.20 a large effect (Cohen, 1973).

## Results

### Behavioral Results

#### Consistency Effect

Participants showed a higher purchase intention in the congruence condition (*M* = 4.303, *S.E*. = 0.098) than in the incongruence condition [*M* = 4.214, *S.E*. = 0.1; *t*_(1,38)_ = 2.117, *p* = 0.041].

#### Conditional Effect

**Figure 2:**
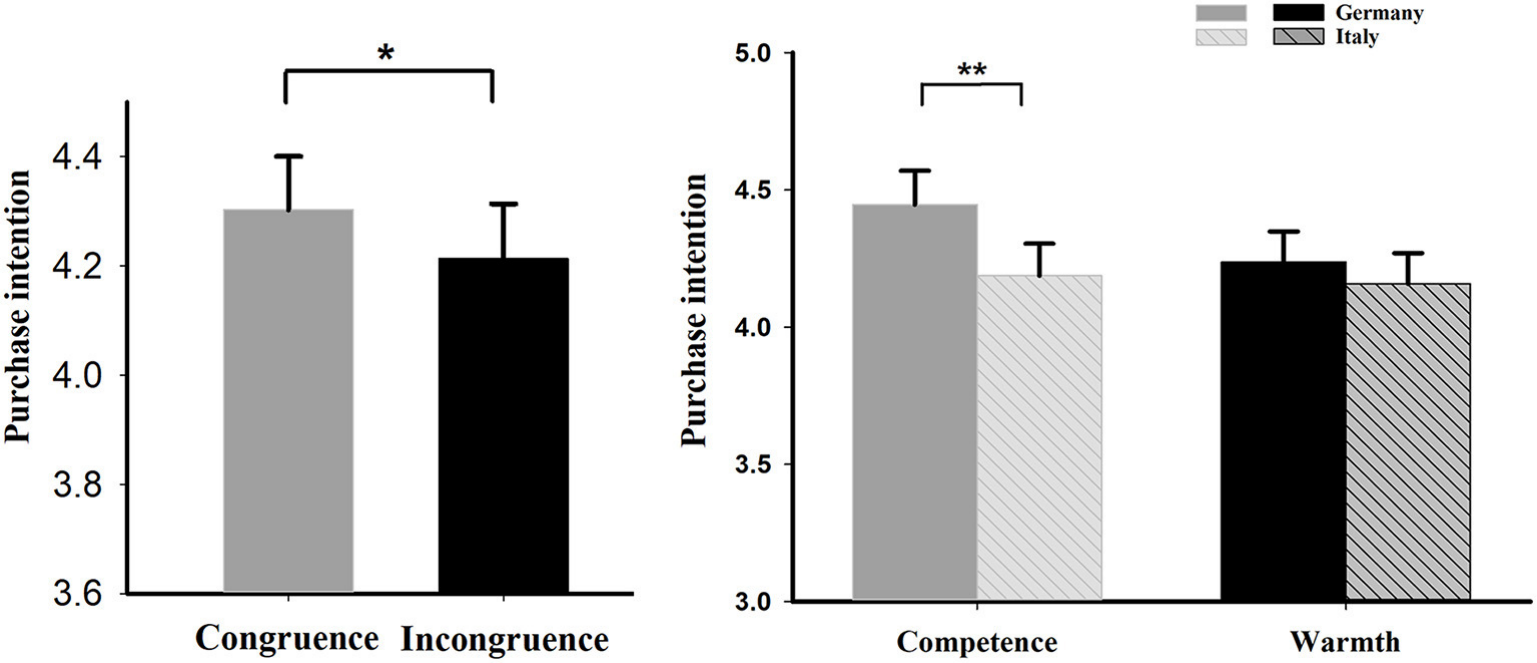
Purchase intention results. *p < 0.05, **p < 0.01.

A two-way 2 (COO stereotypes) × 2 (brand positioning) repeated measure ANOVA was conducted. It showed a significant main effect of COO stereotypes [*F*_(1,38)_ = 8.2538, *p* = 0.007, $\eta_{p}^{2}$ = 0.178], indicating that the consumers' purchase intention for Germany product (*M* = 4.342, *S.E*. = 0.101) is more than Italy (*M* = 4.173, *S.E*. = 0.101) and no significant main effect for brand personality positioning [*F*_(1,38)_ = 1.319, *p* = 0.258, $\eta_{p}^{2}$ = 0.034]. The interaction effect between COO stereotypes and brand personality positioning [*F*_(1,38)_ = 4.481, *p* = 0.041, $\eta_{p}^{2}$ = 0.105] were obvious. Further simple effect analysis indicated that the difference between Germany and Italy was significant under the brand positioning for the competence conditions [*F*_(1,38)_ = 10.850, *p* = 0.002, $\eta_{p}^{2}$ = 0.222], which indicated that the Germany conditions (*M* = 4.447, *S.E*. = 0.123) elicited significantly larger purchase intention than the Italy conditions (*M* = 4.188, *S.E*. = 0.116). However, under the warmth condition, the effect was not significant [*F*_(1,38)_ = 1.441, *p* = 0.237, $\eta_{p}^{2}$ = 0.037]. The purchase intention results were shown in Figure 2.

### Time-Frequency Results

#### Consistency Effect

Participants elicited a larger magnitude of averaged theta-band activity in the incongruence condition (*M* = 0.221 dB; S.E. = 0.055) than in the congruence condition [*M* = 0.079 dB; *S.E*. = 0.056; *t*_(1,38)_ = −2.851, *p* = 0.007].

#### Conditional Effect

**Figure 3:**
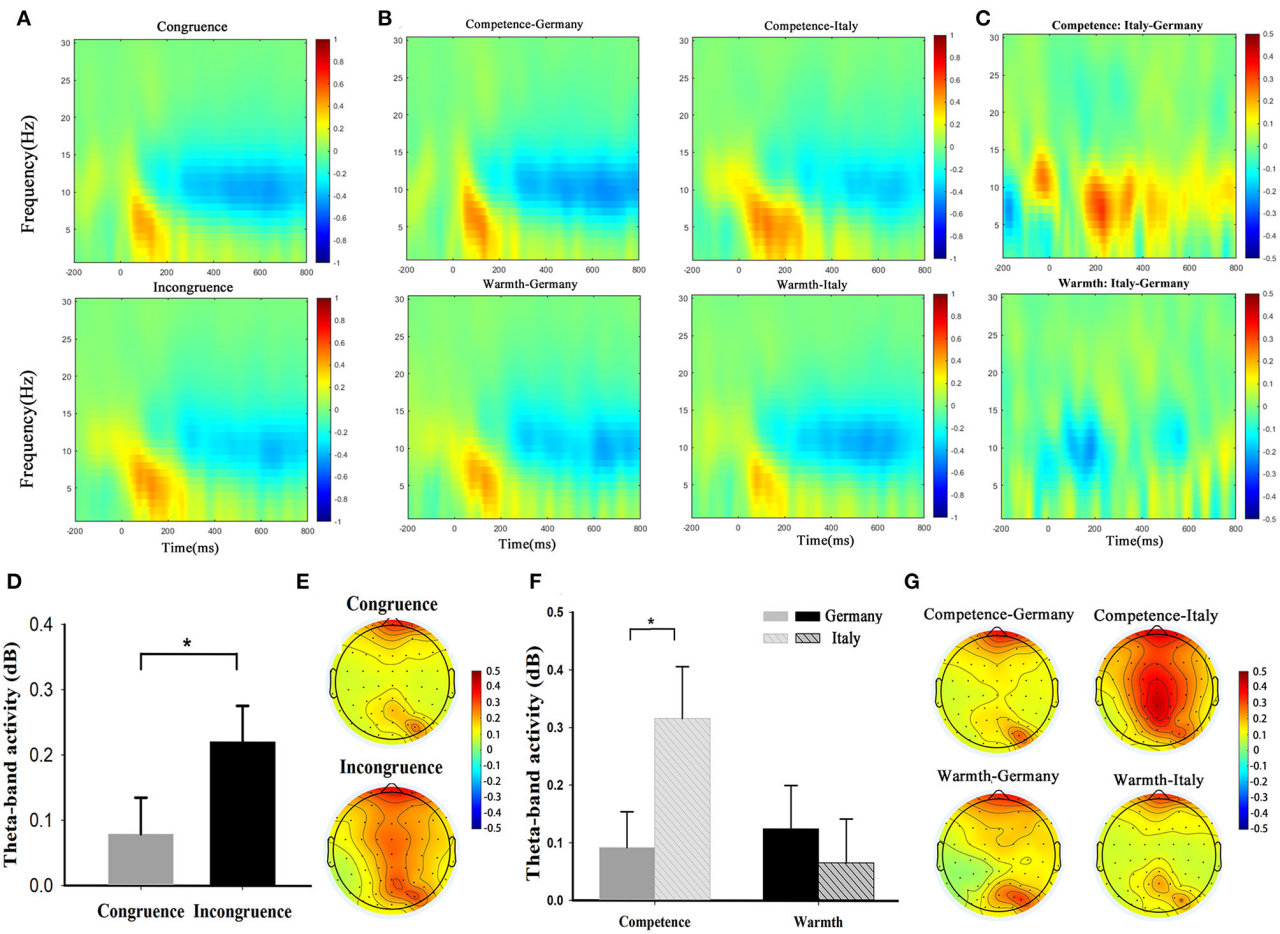
Neural oscillation results. A/B/C Illustration of theta-band oscillations over the frontal ROI under the congruence and incongruence condition **(A)**, four conditions **(B)**, brand positioning difference **(C)**. **(D,F)** Bar graph. **(E,G)** Scalp topographic map. Error bars represent standard error across participants. Asterisk denotes a significant difference. Error bars indicate standard error across participants. *p < 0.05.

We perform a two-way 2 (COO stereotypes) × 2 (brand positioning) repeated measure ANOVA of averaged theta-band activity. The ANOVA result (Figure 3B) showed insignificant main effects of COO stereotypes [*F*_(1,38)_ = 0.904, *p* = 0.348, $\eta_{p}^{2}$ = 0.023] and brand positioning [*F*_(1,38)_ = 3.333, *p* = 0.076, $\eta_{p}^{2}$ = 0.081]. Moreover, we observed a significant interaction between COO stereotypes and brand positioning [*F*_(1,38)_ = 8.130, *p* = 0.007, $\eta_{p}^{2}$ = 0.176]. The further simple effect test indicated participants elicited a larger magnitude of theta-band activity for the Italy (*M* = 0.125 dB, *S.E*. = 0.075) than for the Germany (*M* = 0.092 dB, *S.E*. = 0.062) in the competence brand positioning condition [*F*_(1,38)_ = 4.278, *p* = 0.045, $\eta_{p}^{2}$ = 0.101], However, under the warmth brand positioning condition, the effect was not significant [*F*_(1,38)_ = 0.426, *p* = 0.518, $\eta_{p}^{2}$ = 0.011], results of brain activities are depicted in Figure 3. All the behavioral and neural results are summarized in Table 2.

**Table 2:** The results of behavioral and EEG.

**Behavioral results (Purchase intention)**	**Consistency effect**		**Congruence > Incongruence**
	ME-Country		Germany > Italy
	ME- Brand positioning		N/A
	Interaction effect	Competence condition	Germany > Italy
		Warmth condition	N/A
EEG results (Theta-band)	Consistency effect		Incongruence > Congruence
	ME-Country		N/A
	ME- Brand positioning		N/A
	Interaction effect	Competence condition	Italy > Germany
		Warmth condition	N/A

*N/A means this effect is insignificant. Abbreviations: ME, main effect*.

## Discussion and Conclusion

The purpose of this study is to examine the congruence effect between COO stereotypes and brand positioning on purchase intention, the boundary effect of brand positioning strategy, as well as and the underlying cognitive mechanism.

Behaviorally, in the congruence effect, consumers showed a higher intention to purchase products in the congruent condition than in the incongruent condition. This result is consistent with the previous study (Magnusson et al., 2018), as congruence in brand positioning satisfies consumers' desire for consistency and harmony, therefore facilitates purchase intentions. Moreover, we found that consumers' purchase intention for products from Germany (a competence stereotype country) is higher than for products from Italy (a warmth stereotype country), regardless of the brand positioning. This result suggests that the COO stereotype is critical in shaping consumer perception of international brands (Septianto et al., 2020) and emphasizes the importance of competence in a country stereotype. This is consistent with previous studies, such as reported in Aaker et al. (2012) findings, the competence dimension is a crucial factor driving purchase intention, whereas the warmth dimension is not (Aaker et al., 2012). We also found the conditional effect of purchase intention when the COO stereotypes interacted with the brand positioning strategy. Whereas, the congruence effect shows that congruity enhances consumer purchase intention, the boundary effect evidences that congruity is only accentuated under conditions of competence brand positioning, but not evident in the warmthpositioning condition.

At the brain level, the neural data complemented the behavioral findings and provided additional evidence supporting our primary hypothesis. We first found the frontal theta-band activity was elicited in the COO stereotype-brand positioning incongruence condition compared with the congruence condition. As we noted in the introductory part, the frontal theta oscillation may serve as a marker for cognitive conflict (Nigbur et al., 2011; Cavanagh and Frank, 2014; Liu et al., 2014; Padrão et al., 2015; Brunetti et al., 2019). Thus, our current finding shows that the incongruence elicited higher cognitive conflicts (mirrored by the larger theta oscillations), leading to a sense of resistance, which reduced consumers' purchase intention. According to the cognitive consistency theory, consumers are indeed predisposed to prefer cognitively consistent elements with their existing knowledge (Festinger, 1957). When the structure of the incoming information, i.e., brand positioning, is matched with brain-stored schema of COO, achieving cognitive fit, consumers will experience a relatively simple and easy processing of the brand information, which can result in less cognitive conflicts and favorable outcomes of product evaluation. Secondly, we further found that the theta oscillations were greater for Italian products (than German products) in the competence positioning condition, while this effect was absent in the warmth positioning condition. This result suggests no difference in consumers' perception of the brand's COO, either in terms of competence or warmth stereotypes, under the warmth brands positioning condition. As we pointed out before, the competence dimension provides cues to information that increases the reliability of high-quality products (Wilcox, 2015), and consumers may expect competent brands to stem from competence stereotype country. As a consequence, consumers' cognitive conflict toward competence brands from a warmth stereotype country is higher than those from a competence stereotype country. On the contrary, the warmth dimension is related to consumer satisfaction but not germane to whether a firm will deliver a high-quality offering (Aaker et al., 2010). Moreover, prior study has reported that no amount of warmth compensated in terms of customer satisfaction when the perceptions of brand competence are low (Grandey et al., 2005). Thus, in the context of a warmth brand positioning, there is no difference in the cognitive conflict evoked by a competence stereotype country and by a warmth stereotype country. However, we found that the current results are inconsistent with Kolbl et al. (2020)' study, which concluded that brand warmth has a stronger impact on behavioral outcomes than brand competence. This may be attributable to two reasons: first, their study uses existing brands as research objects, e.g., IKEA and Coca Cola, which have evolved over a long period, and consumers have certain stereotypes about such brands. In our study, we use advertising slogans (no brand name) with different brand positioning to exclude the influence of brand stereotypes. Thus, our study is more applicable to exploring new brand positioning strategies. Second, our research topic is different from theirs. They explore the impact of brand stereotypes (competence/warmth) on consumers' value perceptions, while we focus on the effect of COO stereotypes and brand positioning congruence on consumer behavior and perception. COO stereotypes differ from brand stereotypes in that although they seem to be oversimplified and generalized beliefs, they involve different objects and different mechanisms of formation. Compared with brand stereotypes, COO stereotypes are viewed as a more broad construct based on “representative products, national characteristics, economic and political background, history, and traditions” (Nagashima, 1970). Therefore, our current results may signal those consumers are more willing to accept competent countries to expand more forms of brand positioning.

Our current work makes several critical theoretical contributions. *First*, we established objective and physiological cognitive evidence to support the theory of COO stereotype-brand positioning congruence proposed by Magnusson et al. (2018) from the brain level. That is, by providing neural evidence for cognitive consistency theory in COO stereotype studies, our finding helps to understand how congruence occurs and why it is crucial in brand perception. Specifically, congruence enhances consumers' perception fluency of brand information, reduces cognitive conflict, and ultimately leads to higher purchase intentions. *Second*, given the specific brand positioning strategy, we found a boundary effect in the congruence effect. Our results contribute to the existing COO stereotype literature by suggesting that ignoring specific brand positioning strategies in favor of congruence may lead to poor marketing effects. The COO stereotype-brand positioning congruence effect in international markets does not always work, which depends on a case-by-case analysis. *Practically*, considering the cognitive fluency of consumers' processing of matching country-brand images, global companies should incorporate this into their marketing strategies of brand positioning. More profoundly, multinational companies, especially for firms with warm national stereotypes, can develop more precise marketing strategies to gain comparative advantage in international competition, and find appropriate brand positioning.

Although the present study has offered relevant and exciting insights into how brands should formulate marketing strategies and occupy international markets, there are limitations associated with the research. The first limitation lies in that, the four functional products we select are more affordable for college students as experimental materials. Although this product selection method is widely used in previous studies, these products we used were search products. Several studies posit that product types influence consumer purchase behavior, especially the difference between search and experience products (Nelson, 1974; Korgaonkar et al., 2004). It will be interesting to verify our conclusions from the perspective of experience products. The second limitation is that the laboratory experiment has less external validity, though the internal validity is better (Jimenez-Buedo and Miller, 2010). Future studies on current topics using secondary data or field experiments are therefore encouraged.

In summary, this study adopted EEG technology to investigate the underlying cognitive mechanism behind the COO stereotypes and brand positioning congruence. The behavioral results showed that participants prefer the products whose COO stereotype is congruent with the brand positioning, as opposed to products that are incongruent. The EEG results complemented the behavioral results from the perspective of cognitive conflict. To be specific, consumers elicited stronger cognitive conflict in the incongruence condition than in the congruence condition. In the conditional effect, we further found that consumers prefer products made in a competence stereotype country to those in a warmth stereotype country in the competence positioning condition, but this tendency is indiscernible in the warmth positioning condition. We elaborated our current findings based on the cognitive consistency theory and the cognitive conflict perspective.

## Data Availability Statement

The raw data supporting the conclusions of this article will be made available by the authors, without undue reservation.

## Ethics Statement

The studies involving human participants were reviewed and approved by Academy of Neuroeconomics and Neuromanagement at Ningbo University. The patients/participants provided their written informed consent to participate in this study. Written informed consent was obtained from the individual(s) for the publication of any potentially identifiable images or data included in this article.

## Author Contributions

AW and JJ conceived and designed the study. YL and JL prepared the experimental stimuli and collected the data. AW, DL, and JJ interpreted the data and drafted the manuscript. DL, LG, and JJ reviewed and edited the manuscript. LG and JJ supervised the project. All authors contributed to the article and approved the submitted version.

## Funding

This work was supported by the Ministry of Education of China (Grant No. 21YJAZH035), the China Association for Science and Technology (Grant Nos. 2021ZZZLFZB1207104 and 2021ZZZLFZB1207039), and the National Natural Science Foundation of China (Grant No. 71942002). We thank Xinyu Hu from WFLA for data collection and data analysis.

## Conflict of Interest

The authors declare that the research was conducted in the absence of any commercial or financial relationships that could be construed as a potential conflict of interest.

## Publisher's Note

All claims expressed in this article are solely those of the authors and do not necessarily represent those of their affiliated organizations, or those of the publisher, the editors and the reviewers. Any product that may be evaluated in this article, or claim that may be made by its manufacturer, is not guaranteed or endorsed by the publisher.
